# Enrichment of *Burkholderia* in the Rhizosphere by Autotoxic Ginsenosides to Alleviate Negative Plant-Soil Feedback

**DOI:** 10.1128/Spectrum.01400-21

**Published:** 2021-11-10

**Authors:** Lifen Luo, Luotao Wang, Linmei Deng, Xinyue Mei, Yixiang Liu, Huichuan Huang, Fei Du, Shusheng Zhu, Min Yang

**Affiliations:** a State Key Laboratory for Conservation and Utilization of Bio-Resources in Yunnan, Yunnan Agricultural University, Kunming, Yunnan, China; b Key Laboratory for Agro-Biodiversity and Pest Control of Ministry of Education, Yunnan Agricultural University, Kunming, Yunnan, China; University of Massachusetts Amherst

**Keywords:** microbiome, microbiological degradation, soilborne disease, autotoxicity, plant disease, plant-microbe interactions

## Abstract

The accumulation of autotoxins and soilborne pathogens in soil was shown to be the primary driver of negative plant-soil feedback (NPSF). There is a concerted understanding that plants could enhance their adaptability to biotic or abiotic stress by modifying the rhizosphere microbiome. However, it is not clear whether autotoxins could enrich microbes to degrade themselves or antagonize soilborne pathogens. Here, we found that the microbiome degraded autotoxic ginsenosides, belonging to triterpenoid glycosides, and antagonized pathogens in the rhizosphere soil of Panax notoginseng (sanqi). Deep analysis by 16S rRNA sequencing showed that the bacterial community was obviously changed in the rhizosphere soil and identified the *Burkholderia*-*Caballeronia*-*Paraburkholderia* (BCP) group as the main ginsenoside-enriched bacteria in the rhizosphere soil. Eight strains belonging to the BCP group were isolated, and *Burkholderia* isolate B36 showed a high ability to simultaneously degrade autotoxic ginsenosides (Rb_1_, Rg_1_, and Rd) and antagonize the soilborne pathogen Ilyonectria destructans. Interestingly, ginsenosides could stimulate the growth and biofilm formation of B36, eventually enhancing the antagonistic ability of B36 to *I. destructans* and the colonization ability in the rhizosphere soil. In summary, autotoxic ginsenosides secreted by *P. notoginseng* could enrich beneficial microbes in the rhizosphere to simultaneously degrade autotoxins and antagonize pathogen, providing a novel ecological strategy to alleviate NPSF.

**IMPORTANCE** Autotoxic ginsenosides, secreted by sanqi into soil, could enrich *Burkholderia* sp. to alleviate negative plant-soil feedback (NPSF) by degrading autotoxins and antagonizing the root rot pathogen. In detail, ginsenosides could stimulate the growth and biofilm formation of *Burkholderia* sp. B36, eventually enhancing the antagonistic ability of *Burkholderia* sp. B36 to a soilborne pathogen and the colonization of B36 in soil. This ecological strategy could alleviate NPSF by manipulating the rhizosphere microbiome to simultaneously degrade autotoxins and antagonize pathogen.

## INTRODUCTION

Plant-induced changes in abiotic or biotic properties in the rhizosphere, which in turn influence the performance of the same or other plants, are known as plant-soil feedbacks (PSFs) (1, 2). According to the direction of net effects, PSFs can be classified as positive, neutral, or negative feedback. Negative plant-soil feedbacks (NPSFs) make soil less suitable for the same individuals or other individuals of the same species (3). NPSFs play important roles in maintaining plant diversity and driving community dynamics in natural ecosystems (4, 5), but they are known as important limiting factors of crop productivity in intensive agricultural systems (6). The build-up of harmful pathogens and accumulation of allelopathic autotoxins are the main driving factors promoting NPSF (7–10). Most autotoxins are secreted into the environment to inhibit the performance of the same plant species (11, 12); moreover, some autotoxins have been shown to promote the growth and pathogenicity of pathogens (13–16). Therefore, some autotoxins showed synergistic effects with soilborne pathogens to aggravate NPSF (7, 17).

Based on the mechanism of NPSF, the key strategy to effectively mitigate NPSF is to solve the problems of autotoxins and soilborne pathogens simultaneously. The most obvious counteraction against NPSF is crop rotation or new farmland selection for cultivation. This strategy, however, is no longer possible in many cases, especially in intensive agriculture systems. Another strategy would be resistance breeding, but this strategy is time-consuming and difficult, as the detailed mechanisms of NPSFs are not fully understood. Soil disinfection by heat or chemical means is effective in simultaneously killing soilborne pathogens and degrading autotoxins in soil (18–20), but its wide use is restricted by high costs and environmental problems (21, 22). Therefore, it is urgent to develop a new sustainable management strategy to alleviate NPSF in agricultural systems.

The plant rhizosphere microbiome comprises a highly diverse community of saprotrophic, mutualistic, and pathogenic microbes that can affect plant growth and plant health (23). It is of substantial interest for exploiting beneficial members of plant rhizosphere microbiomes and then using them as biocontrol agents for sustainable management strategies in crop production (24–28). Moreover, it has been mentioned in some studies that microbial communities play important roles in disease suppressiveness, herbicide degradation, pollutant breakdown, and heavy metal transformation (29–31). Whether plants can employ the microbiome to simultaneously degrade autotoxins and antagonize soilborne pathogens, thereby mitigating the harmful effects of NPSF, remains largely obscure.

Sanqi [Panax notoginseng (Burk.) F. H. Chen] is an important medicinal herb (32). Driven by limited land resources and increasing demands, its large-scale cultivation in monoculture systems has led to NPSFs, which have severely hampered its sustainable production. Previous studies have shown that ginsenosides, the main active ingredients of sanqi and belonging to triterpenoid glycosides, in continually cropped soil and root exudates of sanqi could significantly inhibit seed germination and seedling growth (15). In addition, ginsenosides could not only damage the plant cell wall but also promote the growth of the root rot pathogen in the rhizosphere soil, eventually aggravating the occurrence of NPSF due to synergism with the root rot pathogen (16). Therefore, simultaneously degrading autotoxins and suppressing soilborne pathogens to alleviate NPSF is crucial. In this study, we used sanqi as a model crop to (i) confirm whether the microbiome in the rhizosphere soil could degrade autotoxic ginsenosides and antagonize soilborne pathogen and (ii) study whether autotoxic ginsenosides could enrich the ginsenoside-degradative and root rot pathogen-antagonizing microbiome. Based on these studies, we expected to develop a novel ecological strategy to alleviate NPSFs by manipulating the rhizosphere microbiome.

## RESULTS

### The rhizosphere microbiome could suppress the growth of Ilyonectria destructans and degrade ginsenosides.

**FIG 1:**
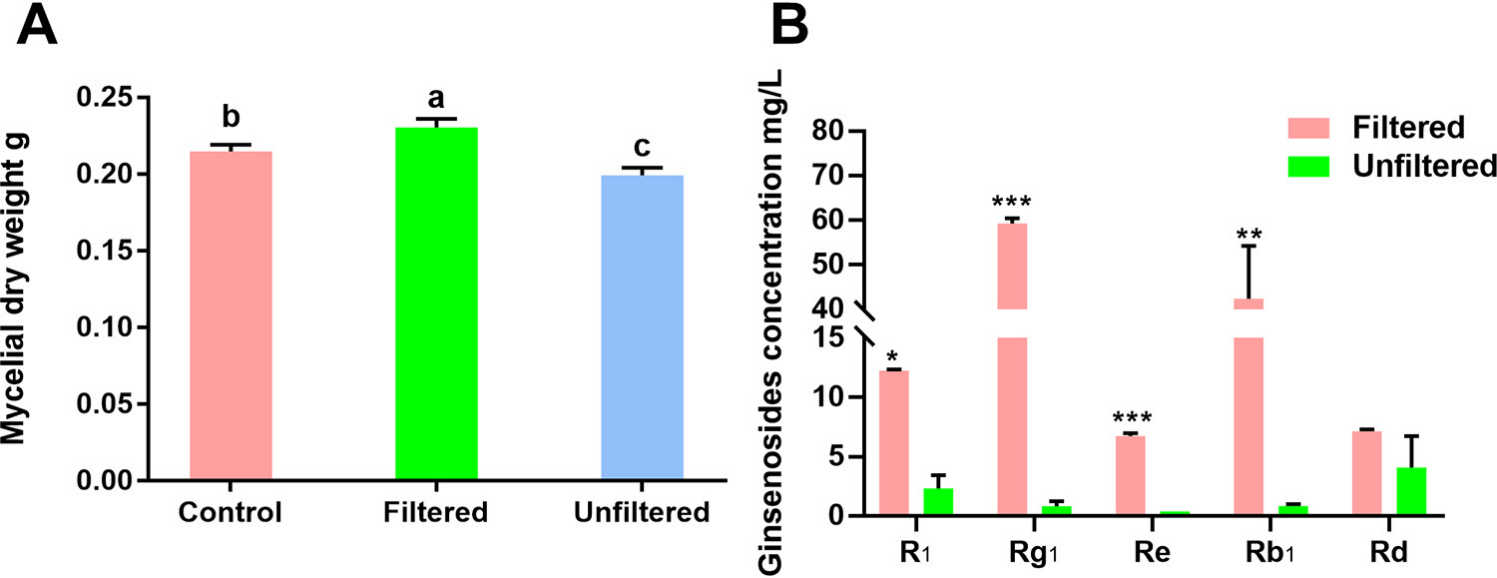
Effects of the rhizosphere microbiome on the growth of *I. destructans* (A) and degradation of ginsenosides (B). Control, *I. destructans* mycelial weight in liquid potato dextrose medium without the soil suspension; filtered, *I. destructans* mycelial weight in liquid potato dextrose medium with rhizosphere soil suspension after filtered with 0.22-μm hydrophilic membranes to remove microbes; unfiltered, *I. destructans* mycelial weight in liquid potato dextrose medium with rhizosphere soil suspension. All values are indicated as the mean ± SE. Bars with different letters indicate significant differences between treatments at a *P* value of <0.05. Asterisks represent significant differences; *, *P* < 0.05; **, *P* < 0.01; ***, *P* < 0.001.

A filtered suspension from the rhizosphere soil amended in medium significantly promoted the growth of *I. destructans* compared with the control, but unfiltered rhizosphere soil significantly suppressed the growth of *I. destructans* (Fig. 1A). Interestingly, unfiltered rhizosphere soil suspension amended in minimal medium showed a significantly high ability to degrade autotoxic ginsenosides, including R_1_, Rg_1_, Rb_1_, and Re. In particular, the concentrations of Rg_1_ and Rb_1_ were reduced by 98.6% and 98.0%, respectively (Fig. 1B).

### Sanqi growth and exogenous ginsenosides modified soil bacterial communities.

**FIG 2:**
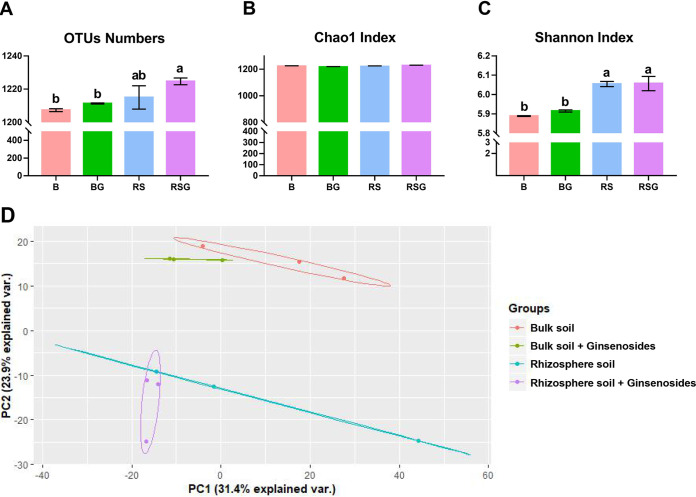
Analysis of the bacterial community in different treated soils based on the OTU level. (A to C) Alpha diversity of different treated soil bacterial communities. (D) Principal-component analysis (PCA) of different treated soil bacterial communities based on the Bray-Curtis distance. B, represents the bulk soil; BG, the bulk soil amended with exogenous ginsenosides; RS, the rhizosphere soil of sanqi; RSG, the rhizosphere soil amended with exogenous ginsenosides. All values are indicated as the mean ± SE. Bars with different letters indicate significant differences between treatments at a *P* value of <0.05.

We analyzed the composition of bacterial communities in the bulk and rhizosphere of sanqi with or without exogenous ginsenosides using 16S rRNA gene amplicon sequencing. A total of 2,084,232 effective bacterial tags were obtained to analyze the change in the bacterial community (see Table S1 in the supplemental material). The number of operational taxonomic units (OTUs) significantly increased in rhizosphere soil with exogenous ginsenosides (Fig. 2A). Although there were no statistically significant differences with respect to the bacterial Chao1 index, the Shannon index in rhizosphere soil with or without exogenous ginsenosides was significantly increased compared with that of their corresponding bulk soil samples (Fig. 2B and C). A principal-component analysis (PCA) showed a clear difference in community composition between bulk and rhizosphere soils with or without exogenous ginsenosides (Fig. 2D). This result demonstrated that plants are the key driving factor of soil microbiota. Exogenous ginsenosides could also modify the soil bacterial community in a certain range, but the effect was not significant (Fig. 2D).

**FIG 3:**
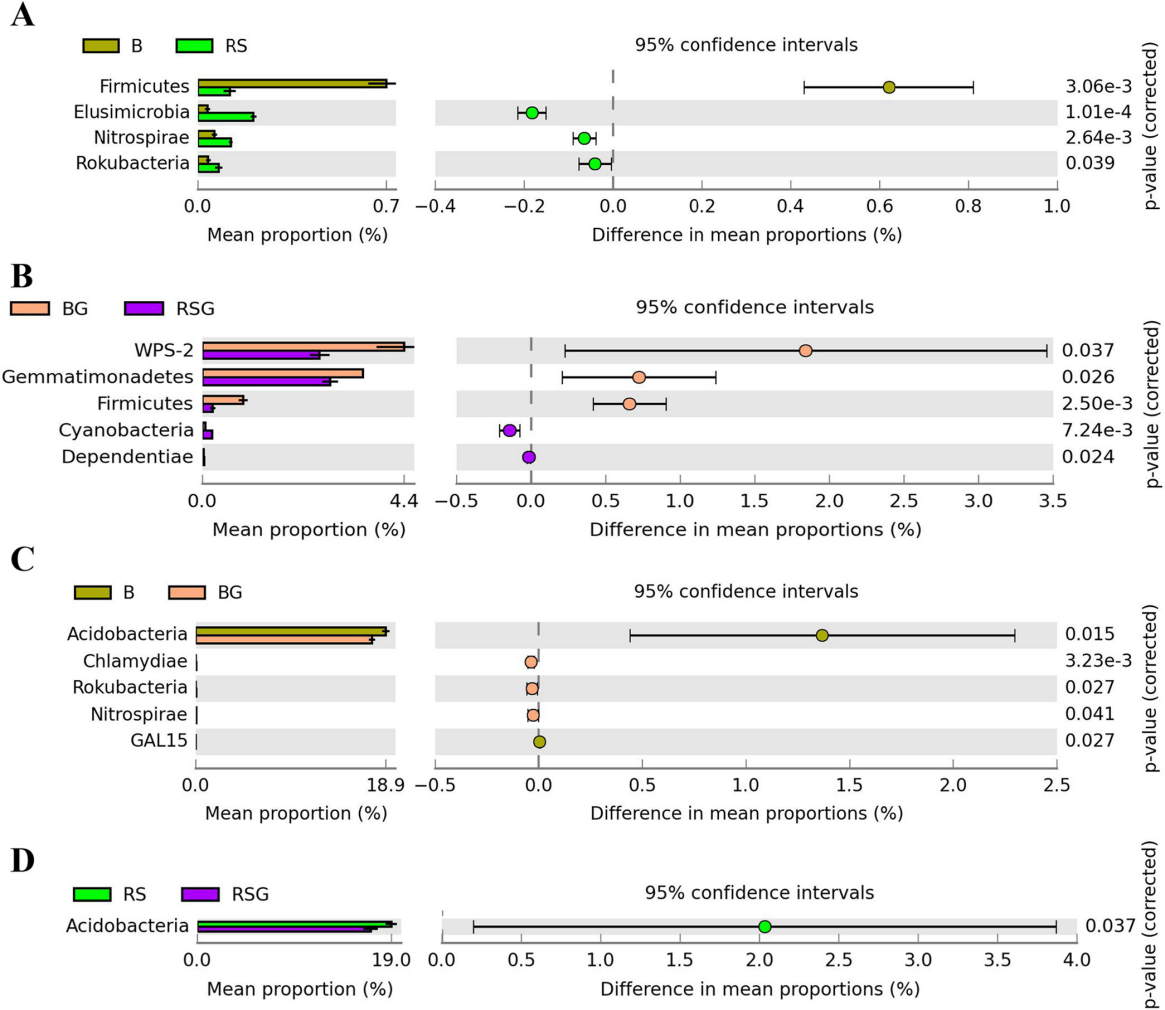
Effects of exogenous ginsenosides on bacteria at the phylum level in rhizosphere and bulk soils. B, the bulk soil; BG, the bulk soil amended with exogenous ginsenosides; RS, the rhizosphere soil of sanqi; RSG, the rhizosphere soil amended with exogenous ginsenosides.

Exogenous ginsenosides altered the relative abundance of some bacteria in bulk and rhizosphere soil at the phylum level (Fig. 3). With the growth of sanqi, the relative abundances of *Elusimicrobia*, *Nitrospirae*, and *Rokubacteria* were significantly enriched in the rhizosphere soil compared with those in bulk soil, but the relative abundance of *Firmicutes* was significantly suppressed (Fig. 3A). When sanqi was grown in soil amended with ginsenosides, the relative abundances of *Cyanobacteria* and *Dependentiae* were significantly enriched, but the relative abundances of WPS-2, *Gemmatimonadetes*, and *Firmicutes* were significantly suppressed in rhizosphere soil compared with those in bulk soil (Fig. 3B). For bulk soil, exogenous ginsenosides could significantly enrich the relative abundances of *Chlamydiae*, *Nitrospirae*, and *Rokubacteria* but suppress the relative abundances of *Acidobacteria* and GAL15 (Fig. 3C). Consistently, exogenous ginsenosides also suppressed the enrichment of *Acidobacteria* in rhizobacterial taxa compared with rhizosphere soil without exogenous ginsenosides (Fig. 3D).

**FIG 4:**
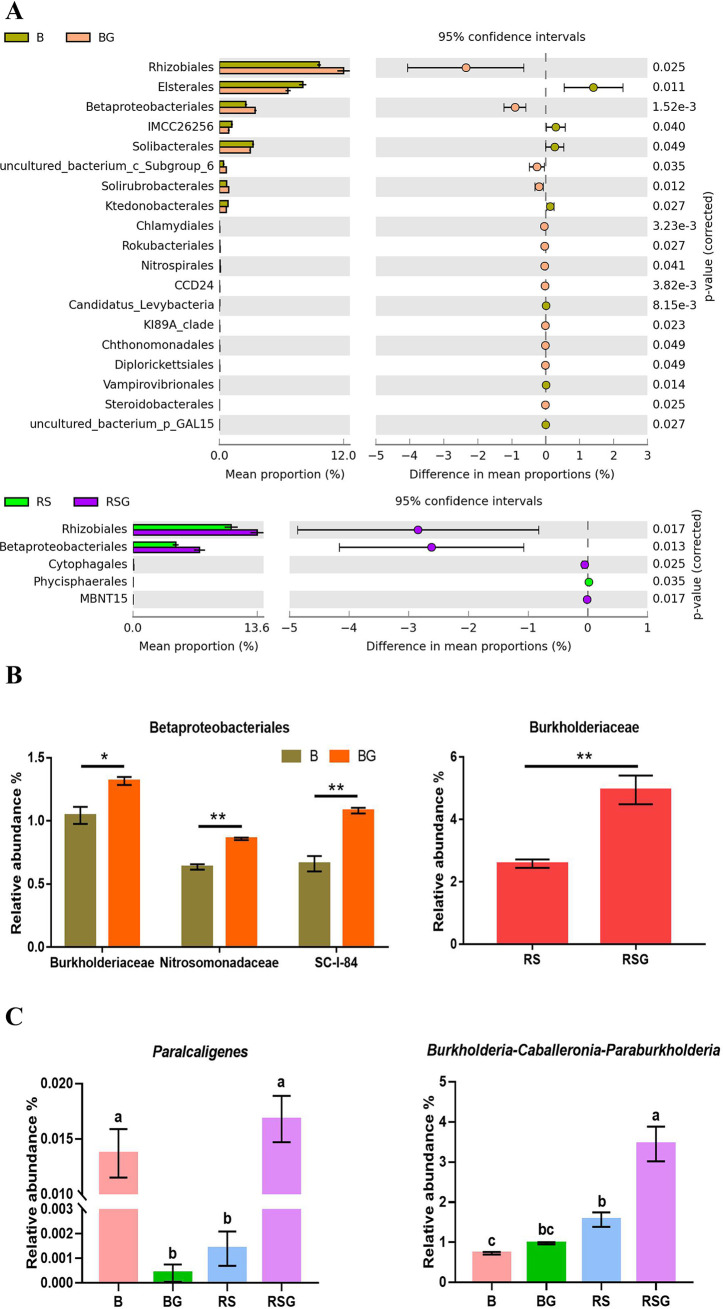
Sanqi recruited specific microbes in the rhizosphere soil when stressed by autotoxic ginsenosides at the order (A), family (B), or genus level (C). B, the bulk soil; BG, the bulk soil amended with exogenous ginsenosides; RS, the rhizosphere soil of sanqi; RSG, the rhizosphere soil amended with exogenous ginsenosides. All values are indicated as the mean ± SE. Bars with different letters indicate significant differences between treatments at a *P* value of <0.05. Asterisks represent significant differences; *, *P* < 0.05; **, *P* < 0.01.

Further analysis demonstrated that 19 orders were significantly changed in bulk soil amended with ginsenosides, and a total of 5 orders were significantly changed in rhizosphere soil amended with exogenous ginsenosides (Fig. 4A). Among them, *Rhizobiales* and *Betaproteobacteriales* were enriched in the relative abundance of exogenous ginsenosides in bulk and rhizosphere soils (Fig. 4A). In *Rhizobiales*, 16 families were detected, of which 3, namely, *Methyloligellaceae*, *Rhodomicrobiaceae*, and *Xanthobacteraceae*, were significantly enriched in bulk soil amended with ginsenosides, and *Rhizobiaceae* was significantly enriched by exogenous ginsenosides in rhizosphere soil (see Table S2 in the supplemental material). Eleven families in *Betaproteobacteriales* were detected. Among them, *Burkholderiaceae* were significantly enriched by exogenous ginsenosides in both bulk and rhizosphere soil (Fig. 4B; see Table S3 in the supplemental material). Thirty genera in *Burkholderiaceae* were detected, of which 8 were significantly enriched in bulk soil and 6 were significantly enriched in rhizosphere soil with exogenous ginsenosides (see Table S4 in the supplemental material). It is worth mentioning that the relative abundance of the *Paralcaligenes* and *Burkholderia*-*Caballeronia*-*Paraburkholderia* (BCP) group was significantly changed by exogenous ginsenosides in bulk and rhizosphere soil (Table S4). Among them, the relative abundance of BCP was significantly increased in the rhizosphere soil compared with that in bulk soil. In particular, the relative abundance of BCP was largely increased in the rhizosphere soil amended with exogenous ginsenosides (Fig. 4C; Table S4).

### BCP isolates showed degradative activity to autotoxic ginsenosides.

**FIG 5:**
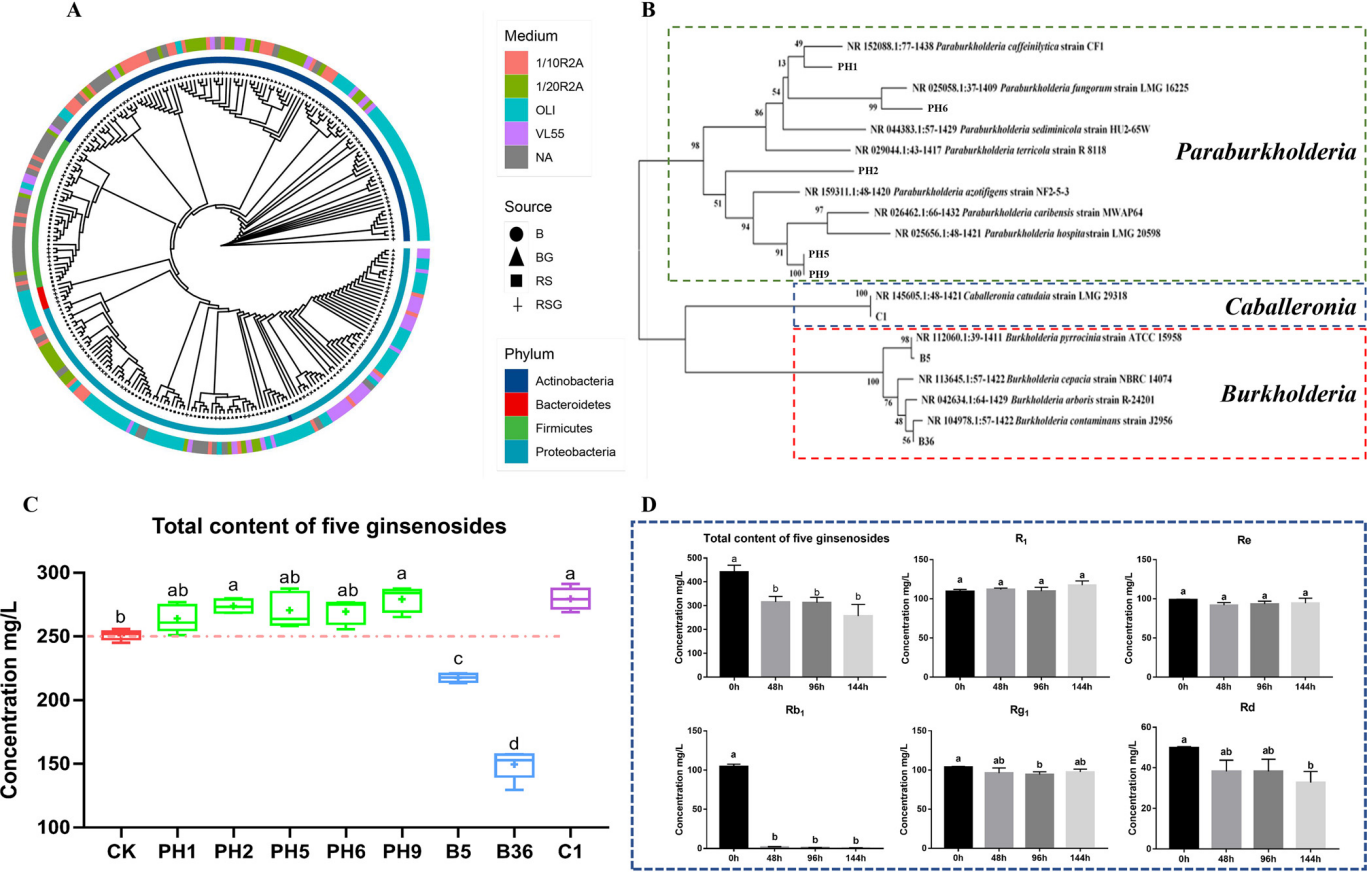
Isolation and identification of rhizosphere microorganisms and degradative activity of the BCP isolates against autotoxic ginsenosides. (A) Phylogenetic tree of 251 isolates based on 16S rRNA genes sequences. Inner circles of different colors represent the phylum level, and the different colors in the outer circle represent the different mediums of isolates. B, the bulk soil; BG, the bulk soil amended with exogenous ginsenosides; RS, the rhizosphere soil of sanqi; RSG, the rhizosphere soil amended with exogenous ginsenosides. (B) Phylogenetic tree of eight isolates belonging to *Burkholderia*-*Caballeronia*-*Paraburkholderia* (BCP) based on 16S rRNA sequences. (C) Total contents of the five main ginsenosides in the solution after inoculation with BCP for 144 h. (D) The degradation of five ginsenosides in medium by isolate B36. All values are indicated as the mean ± SE. Bars with different letters indicate significant differences between treatments at a *P* value of <0.05.

In order to verify whether the BCP group had the ability to degrade autotoxic ginsenosides, we isolated culturable bacteria from the bulk and rhizosphere soils with or without exogenous ginsenosides. A total of 251 bacterial isolates were isolated and phylogenetically characterized (Fig. 5A). These isolates represented four phyla, mostly *Actinobacteria* and *Proteobacteria*, followed by *Firmicutes* and *Bacteroidetes* (Fig. 5A). At the genus level, the top five genera with isolation frequencies were *Microbacterium* (17.9%), *Achromobacter* (17.1%), *Leifsonia* (12.4%), *Bacillus* (9.56%), and *Ochrobactrum* (4.78%) (Fig. 5A). Furthermore, eight isolates were identified as belonging to the BCP group (Fig. 5A and B), of which two were identified as belonging to the genus *Burkholderia* (B5 and B36), one was identified as belonging to the genus *Caballeronia* (C1), and five were identified as belonging to the genus *Paraburkholderia* (PH1, PH2, PH5, PH6, and PH9) according to the phylogenetic analysis (Fig. 5A and B).

We further examined the ability of these eight isolates (PH1, PH2, PH5, PH6, PH9, B5, B36, and C1) that belonged to BCP group to degrade ginsenosides in minimal medium (Fig. 5C). Among them, two *Burkholderia* spp. (B5 and B36) showed a significant ability to degrade ginsenosides in the medium. Particularly, B36 had the highest ginsenoside-degradative ability compared with the other isolates (Fig. 5C and D). Specifically, B36 degraded ginsenosides Rb_1_, Rg_1_, and Rd (Fig. 5D). The concentration of Rb_1_ was significantly decreased at 48 h, and the degradation rate reached 98.29% (Fig. 5D). The concentration of Rg_1_ was significantly decreased at 96 h with a degradation rate of 7.35% (Fig. 5D). The concentration of Rd was significantly decreased at 144 h, with a degradation rate of 34.29% (Fig. 5D).

### *Burkholderia* sp. B36 showed a high antagonistic and colonization ability in ginsenoside-amended media and soil.

**FIG 6:**
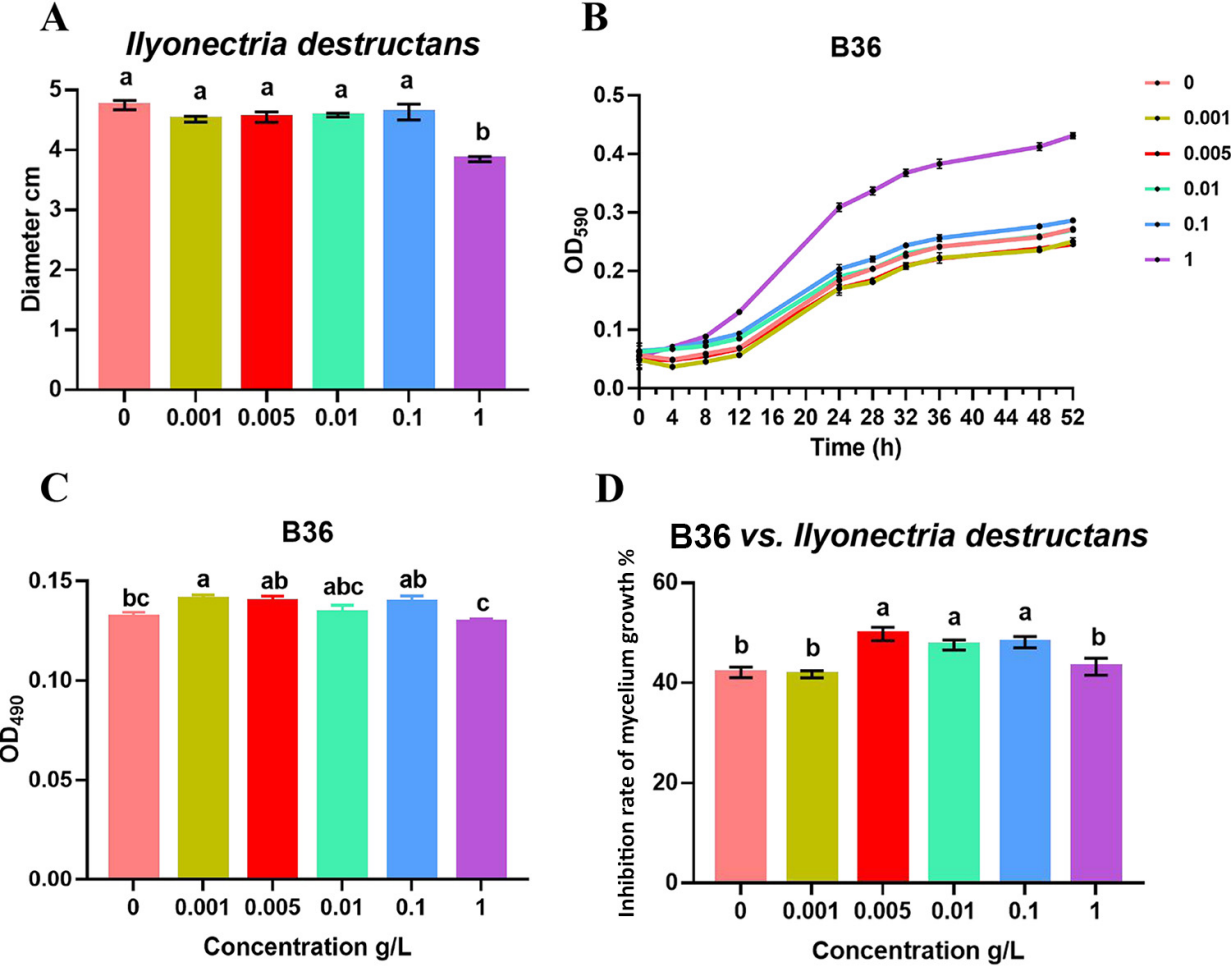
Effects of ginsenosides on the growth of *Ilyonectria destructans* (A) or B36 (B), as well as the biofilm formation (C) and antagonistic activity of B36 against *I. destructans* (D). All values are indicated as the mean ± SE. Bars with different letters indicate significant differences between treatments at a *P* value of <0.05.

The mycelial growth of *I. destructans* was not significantly inhibited by ginsenosides until the ginsenoside concentration reached 1.0 g/liter (Fig. 6A). The growth of B36 cells was promoted with time when they were cultured in 1/10 LB medium containing different concentrations of ginsenosides compared with the blank control (Fig. 6B). In particular, the growth of B36 cells was significantly stimulated by ginsenosides at a concentration of 1.0 g/liter (Fig. 6B). In addition, the biofilm formation of B36 was promoted by ginsenosides at concentrations from 0.001 to 0.1 g/liter (Fig. 6C).

We tested the antagonistic ability of B36 against the mycelial growth of *I. destructans* on media with and without exogenous ginsenosides. B36 showed obvious antagonistic activity against the growth of *I. destructans* without ginsenosides, while it showed enhanced antagonistic activity when medium was amended with ginsenosides at concentrations of 0.005, 0.01, and 0.1 g/liter (Fig. 6D).

**FIG 7:**
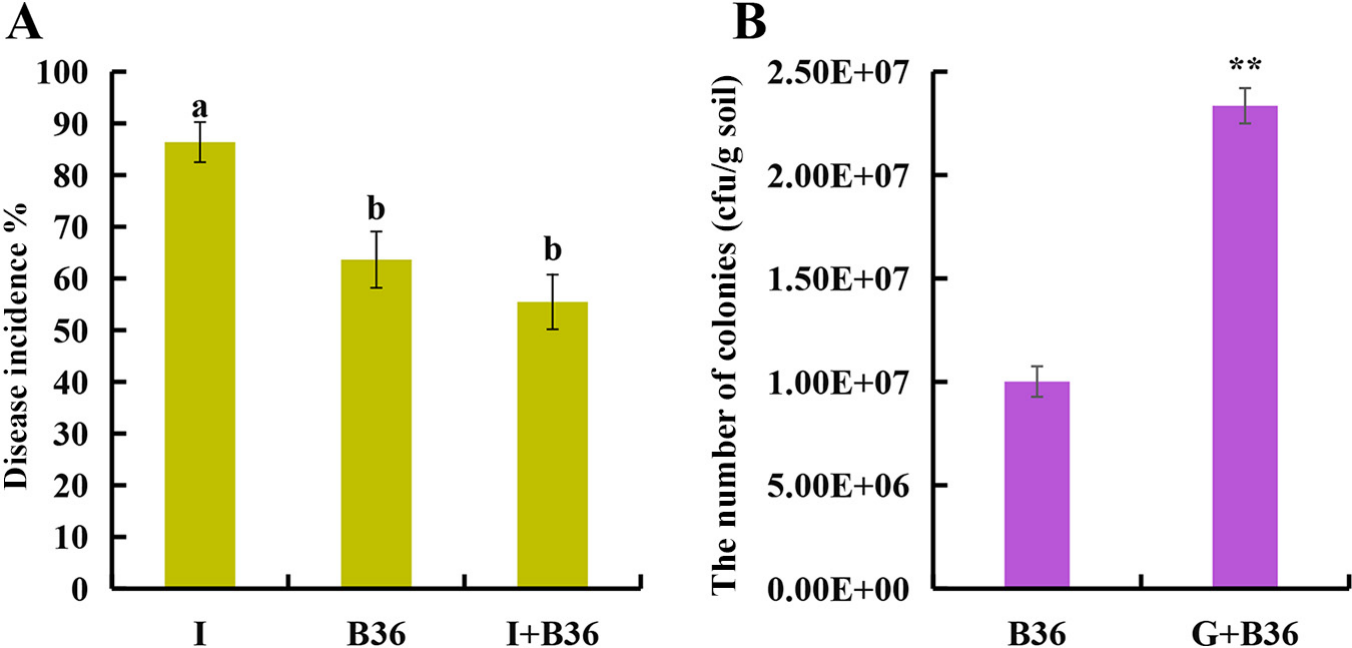
Effects of ginsenosides on the ability of B36 to control soilborne disease and colonize soil. (A) Effects of *Burkholderia* sp. B36 on the incidence rate of sanqi in the soil with or without *I. destructans*. (B) Colonization of B36 in rhizosphere soils of sanqi with or without exogenous ginsenosides. I, the sterilized natural soil inoculated with *I. destructans*; B36, the sterilized natural soil inoculated with B36; I+B36, the sterilized natural soil inoculated with *I. destructans* and B36; G+B36, the rhizosphere soil with ginsenosides inoculated with B36. All values are indicated as the mean ± SE. Bars with different letters indicate significant differences between treatments at a *P* value of <0.05. Asterisks represent significant differences; **, *P* < 0.01.

Further experiments demonstrated that B36 could decrease the incidence rate of sanqi compared with that of pathogen *I. destructans* inoculation in sterilized soil (Fig. 7A). B36 decreased the incidence rate of sanqi even though the soilborne pathogen *I. destructans* was synergistically inoculated with B36 in the soil (Fig. 7A). In addition, B36 could colonize the rhizosphere soil after 3 months of inoculation, and the colonization ability was enhanced in the rhizosphere when the soil was amended with ginsenosides (Fig. 7B).

## DISCUSSION

Plants harbor a variety of microorganisms in the rhizosphere that in turn have an obvious effect on the development and fitness of plants. Autotoxicity and soilborne disease, the main agents of NPSF, have lead to dramatic crop yield losses and quality decline (7, 16). In this study, we found that the rhizospheric soil bacterial community was changed by sanqi, especially BCP group, which were significantly enriched in the rhizosphere when sanqi were grown in soil with exogenous ginsenosides. *Burkholderia* sp. B36 isolated from the BCP group could simultaneously degrade autotoxic ginsenosides and antagonize a soilborne pathogen. Importantly, the antagonistic ability of B36 against the growth of a pathogen and the colonization ability of B36 in soil could be enhanced by ginsenosides through stimulation of growth and biofilm formation.

The rhizosphere is the dynamic and complex interface between the plant root and soil (33). Root exudates have been recognized as the main driving factor of soil microbial community dynamic change or assembly and provide the main carbon and energy sources for the rhizosphere microbiome (14, 34–36). Ginsenosides, widely identified in the root exudates and the rhizosphere soil of *Panax* genus plants, could modify the community of the microbiome in the rhizosphere soil (10, 15, 37, 38). In this study, we found that the growth of sanqi with or without exogenous ginsenoside could significantly modify the structure of microbial communities in the rhizosphere soil (Fig. 3A and B), consistent with previous studies showing that sanqi growth suppressed the abundance of *Acidobacteria* and *Firmicutes* (10). Further analysis found that exogenous ginsenosides could significantly suppress *Acidobacteria* but enrich *Nitrospirae* in both bulk and rhizosphere soils (Fig. 3C and D). However, only *Burkholderiaceae* at the family level were significantly enriched by exogenous ginsenosides in bulk and rhizosphere soil (Fig. 4B; Table S3). At the genus level, the relative abundance of *Paralcaligenes* and BCP in *Burkholderiaceae* was significantly changed by exogenous ginsenosides in bulk and rhizosphere soil (Fig. 4C; Table S4). However, *Paralcaligenes* was enriched by exogenous ginsenosides in rhizosphere soil but suppressed by exogenous ginsenosides in bulk soil (Fig. 4C; Table S4). Only the relative abundance of BCP in Burkholderiaceae was significantly enriched in the bulk soil with exogenous ginsenosides (Table S4) and rhizosphere soil (Fig. 4C), and the relative abundance of BCP was further enriched in the rhizosphere soil by exogenous ginsenosides (Fig. 4C). These data validated that the enrichment of BCP was driven by exogenous ginsenosides (Fig. 4).

The BCP group is composed of the three independent genera *Burkholderia*, *Caballeronia*, and *Paraburkholderia*. Due to their close genetic relationship, the V3+V4 segment of 16S rRNA selected in this sequencing failed to separate the specific genus (39, 40). Among these three genera, *Burkholderia* was reported as one kind of plant growth-promoting rhizobacteria and is widely found in the environment and rhizosphere, generally promoting plant growth and improving plant resistance or alleviating toxicity from the glyphosate or benzoic acid and phthalic acid (41–46). Here, we found that microbiota in the rhizosphere soil could not only suppress the growth of *I. destructans* but also degrade autotoxic ginsenosides (Fig. 1A and B). Based on the above ginsenoside-enriched BCP analysis, we isolated eight isolates from BCP (Fig. 5B) and found that only isolates from *Burkholderia* showed the ability to simultaneously degrade autotoxins and antagonize a pathogen (Fig. 5C and D, Fig. 6D). Among them, the *Burkholderia* sp. B36 showed a significant ability to decrease ginsenosides (Rb_1_, Rg_1_, and Rd) (Fig. 5C and D), which were reported to cause autotoxicity against root cell growth (15). These data demonstrated that ginsenoside could enrich *Burkholderia* sp. to alleviate autotoxicity by degradation. The degradation of ginsenosides may be due to the hydrolyzation of *Burkholderia* sp. to ginsenosides. A previous study demonstrated that ginsenosides could be hydrolyzed by microbial glycosyl hydrolases to release glycosyl groups as nutrients for microbes (47). For example, Phytophthora cactorum could not only detoxify ginsenosides through detoxification-related enzymes but also utilize them as nutrients for growth by glycosyl hydrolases (16). Additionally, there are many bacteria, including Nocardioides panaciterrulae sp. nov. and Flavisolibacter ginsenosidimutans sp. nov., reported to have the ability to transform the dominant ginsenosides to rare active components (48, 49). Moreover, ginsenosides in root exudates of sanqi could be used as a carbon source to drive changes in soil microbiota (50), and our study demonstrated that ginsenosides could stimulate the growth of B36 (Fig. 6B). These data confirmed that B36 could hydrolyze or converse ginsenosides and then act as a carbon for growth, but the underlying mechanism should be further identified.

*Burkholderia* sp. B36 showed strong antagonistic activity against the soilborne pathogen *I. destructans* (Fig. 6D). Previous research showed that plant growth-promoting bacteria, such as *Burkholderia* sp., Pseudomonas sp., and *Bacillus* sp., inoculated into continuously cultivated soil could significantly alleviate NPSF and the occurrence of crop root rot (10, 51–53). Our pot experiment also supported that *Burkholderia* sp. B36 decreased the incidence rate of sanqi and alleviated the infection of the soilborne pathogen *I. destructans* (Fig. 7A). Interestingly, B36 showed higher antagonistic activity against *I. destructans* on media amended with ginsenosides compared with that of media without ginsenosides (Fig. 6D) and a higher colonization ability (Fig. 7B) when soil was amended with ginsenosides. The antagonistic mechanism employed by *Burkholderia* sp. and other PGPRs includes mainly the secretion of antibacterial substances, the competition of ecological niches and nutrients, and the promotion of growth (54–57). The stimulation of ginsenosides to the growth of the B36 isolate may be one kind of mechanism (Fig. 6B). However, previous studies demonstrated that ginsenosides could promote the growth of pathogens and enhance the expression of virulence factors of pathogens (13, 15, 16). Whether ginsenoside stress could promote the secretion of antagonistic substances to enhance the antagonistic activity of B36 against the root rot pathogen deserves further study. In addition, previous studies demonstrated that biofilms play an important role in the colonization of bacteria and enhance the competition of bacteria (58–60). The high colonization ability of B36 in the rhizosphere soil maybe due to the stimulation of ginsenosides to the growth and biofilm formation of the B36 isolate (Fig. 6C).

Therefore, autotoxic ginsenosides could directly enrich *Burkholderia* sp. in the rhizosphere soil to degrade autotoxins and antagonize the root rot pathogen. In addition, plants can indirectly recruit beneficial microbes to help themselves or their offspring cope with biotic and abiotic stresses by changing root exudates (61–64). Our previous studies demonstrated that autotoxic ginsenosides could stress root cells and cause root cell death by inducing the overaccumulation of reactive oxygen species (15, 16). However, whether autotoxic ginsenosides could induce changes in root exudates and then recruit microbiota to reduce the harm of these substances to plant growth is still unknown and deserves further identification.

In conclusion, autotoxic ginsenosides, secreted by sanqi into soil, could enrich *Burkholderia* sp. to alleviate NPSF by degrading autotoxins and antagonizing the root rot pathogen. In detail, ginsenosides could stimulate the growth and biofilm formation of B36, eventually enhancing the antagonistic ability of *Burkholderia* B36 to the soilborne pathogen and the colonization of B36 in soil. This is a novel ecological strategy to alleviate NPSF by manipulating the rhizosphere microbiome to simultaneously degrade autotoxins and antagonize a pathogen.

## MATERIALS AND METHODS

### Measurement of the ability of the sanqi rhizosphere soil microbiome to suppress the growth of a pathogen or degrade ginsenosides.

Rhizosphere soil, collected from sanqi grown for 3 years in July 2017 from Xundian County, Yunnan, China (103.13°E, 25.67°N; altitude of 1,960 m), was used to test whether the rhizospheric soil contained microbes to suppress the growth of *Ilyonectria destructans* and degrade autotoxic ginsenosides according to previous methods with some modifications (65). Briefly, 100 grams of each soil sample was added to 900 ml of sterilized water and rotated for 30 min (25°C, 12,000 × *g*) after homogenization. The suspensions were filtered with qualitative filter paper and then divided into the following two subsamples: one subsample was filtered with 0.22-μm hydrophilic membranes to remove microbes and the other subsample was not treated. To test the antifungal ability of the soil suspension, 5 *I. destructans* mycelium blocks (6-mm diameter) were added to 45 ml of potato dextrose fluid medium (containing 200 g of potato and 20 g of glucose in 1 liter of water), which was shaken at 150 rpm for 72 hours. Five milliliters of each treated soil suspension was added to the medium and then continuously shaken at 150 rpm for 72 hours. Medium containing 5 ml of water was used as a control, and six replicates were used for each treatment. Then, all treatments were filtered with qualitative filter paper and dried, and the hyphae were weighed.

To test the degradation ability, a total of 500 μl of each treated soil suspension was added to 50 ml of minimal medium (3 g of NaNO_3_, 1 g of K_2_HPO_4_, 0.5 g of MgSO_4_·7H_2_O, 0.5 g of KCl, and 0.01 g of FeSO_4_ in 1.0 liter of distilled water) containing 1.0-g/liter crude ginsenosides and rotated on a shaker at 150 rpm for 72 h (25°C). Samples (2.0 ml) were concentrated to dryness under reduced pressure for analysis by high-performance liquid chromatography (HPLC). The ginsenosides in minimal medium were identified and quantified using HPLC according to a previous study (9). Chromatographic separation was performed on an Agilent Poroshell 120 EC-C_18_ column (4.6 mm by 150 mm, 4 μm). The solvent system included solvent A (acetonitrile) and solvent B (water). The following multistep gradient program was implemented: 0 to 20.0 min, 82% B; 20.0 to 40.0 min, 82% to 57% B; 40.0 to 48.0 min, 57% to 45% B; 48.0 to 54.0 min, 45% B; 54.0 to 56.0 min, 45% to 5% B; 56.0 to 71.0 min, 5% B; 71.0 to 72.0 min, 5.0% to 82% B; and 72.0 to 74.0 min, 82% B. An injection volume of 10 μl and a flow rate of 1.0 ml/min were utilized. The column temperature was maintained at 30°C, and data were acquired at 203 nm. The target compounds in minimal medium were identified by comparing their retention times to those of ginsenoside standards Re (CAS number 51542-56-4), Rg_1_ (CAS number 22427-39-0), Rd (CAS number 52705-93-8), Rb_1_ (CAS number 41753-43-9), and R_1_ (CAS number 80418-24-2) (purity, ≥98%), which were purchased from Guizhou Dida Biological Technology Co. (Guizhou, China). The content of the target compounds in minimal medium was quantified using standard curves that showed the linear relationships between the peak areas and the concentrations.

### Effect of exogenous ginsenosides on the rhizospheric microbiome.

### Experimental design.

Natural soil, used to estimate the influences of ginsenosides on the rhizospheric soil microbiome, was collected from a pine forest in November 2017 in Xundian County, Yunnan, China (103.13°E, 25.67°N; altitude of 1,960 m). The surface of the soil (top 10 to 15 cm) was removed, and the layer between 15 and 30 cm was collected as natural soil. The natural soil was sieved twice (5- and 2-mm mesh) to remove plant residues in the laboratory. A 90-g portion of soil was transferred into a glass bottle (250 ml); treated with 10 ml of diluted crude ginsenosides containing R_1_, Rg_1_, Rb_1_, Re, and Rd according to the concentrations in consecutively cultivated soil (15); and then maintained for 2 days to ensure ginsenosides were distributed evenly in the soil. The soil watered with distilled water was set as the blank control. The bottles with soils treated with crude ginsenosides or distilled water were divided into two parts. Sanqi seeds surface sterilized with 1% sodium hypochlorite were placed in the soils of half of the bottles, and 10 seeds were placed in the soil of each bottle. Each treatment was repeated nine times using a randomized design. All treatments were incubated in a programmable illuminated incubator with a light/dark (L/D) cycle of 12 h/12 h and a temperature cycle of 25°C/20°C (day/night).

### Soil sampling.

After 4 weeks of sanqi seed emergence, the seedlings were uprooted, and the rhizosphere soil was collected following the procedure used previously for *Arabidopsis* sp. with some modifications (66). We removed loose soil on all root surfaces until the remaining aggregates were within this range. Roots were placed in a clean and sterile 50-ml tube containing 40 ml of 1× phosphate-buffered saline (PBS) buffer. Tubes were vortexed at maximum speed for 15 seconds, which released most of the rhizosphere soil from the roots. The washing buffer was subjected to centrifugation (12,000 × *g* for 10 min), and the collected precipitate was defined as rhizosphere soil. No planted soil was collected as the bulk soil. The soils from all treatments were collected in triplicate and divided into two subsamples, as follows: one subsample was stored at −80°C for future use, and the other was kept at 4°C for culturable bacterial isolation.

### Sequence analysis of the 16S rRNA genes.

Total soil genomic DNA was extracted from the above soil samples using the PowerSoil DNA isolation kit (Mi Bio Laboratories, Inc., Carlsbad, CA, USA) according to the manufacturer's instructions. DNA concentration and purity were quantified using a NanoDrop 2000 spectrophotometer (Thermo Fisher Scientific, USA). Purified DNA was stored at −80°C for future use.

Bacterial 16S rRNA genes in the rhizosphere soil total DNA samples were sequenced using the Illumina HiSeq platform of the Novogene Corporation (Beijing, China). The bacterial genes were amplified with the primers 5′-ACTCCTACGGGAGGCAGCA-3′ and 5′-GGACTACHVGGGTWTCTAAT-3′ (16S rRNA V3-V4 region gene) (67). Sequenced reads were spliced using FLASH (v1.2.7) (68) and were quality filtered (69). Sequences were assembled for each sample according to the unique barcode using QIIME (70). Through quality filtering and chimera removal, the retained effective tags were used to perform operational taxonomic unit (OTU) clustering and species annotation. OTUs were defined at ≥97% sequence identity using UCLUST (70). The taxonomic identities of the bacteria were determined using Silva schemes (71). Finally, the data of each sample were processed by normalization based on the minimum data in the sample, and then community richness and diversity and principal-coordinate analysis (PCoA) were conducted.

### Isolation and identification of BCP microbiota.

One gram of each soil sample was collected from the above treatments and added to 9.0 ml of 0.1% (wt/vol) solution of sodium pyrophosphate. After homogenization for 15 min, the soil suspension was serially diluted (10^−3^ to 10^−5^) and 100 μl of the solutions was plated on beef extract-peptone (NA) medium (containing 10.0 g of triptone, 3.0 g of beef-extract, 10.0 g of sodium chloride, and 15.0 g of agar 1 liter of pH 7.4 to 7.6 water) (72), 1/10 R2A (containing 0.05 g of casein acid hydrolysate, 0.05 g of yeast extract, 0.05 g of proteose peptone, 0.05 g of dextrose, 0.05 g of starch, 0.3 g of dipotassium hydrogen phosphate, 0.024 g of magnesium sulfate, 0.3 g of sodium pyruvate, and 15.0 g of agar in 1 liter of pH 7.2 water), 1/20 R2A (containing 0.025 g of casein acid hydrolysate, 0.025 g of yeast extract, 0.025 g of proteose peptone, 0.025 g of dextrose, 0.025 g of starch, 0.3 g of dipotassium hydrogen phosphate, 0.024 g magnesium sulfate, 0.3 g of sodium pyruvate, and 15.0 g of agar in 1 liter of pH 7.2 water), OL1 (containing 0.5 g of magnesium sulfate, 0.5 g of potassium nitrate, 1.3 g of dipotassium hydrogen phosphate, 0.06 g of calcium nitrate, 0.05 g of dextrose, 0.004 g of casein acid hydrolysate, and 15.0 g of agar in 1 liter of pH 7.2 water), and VL55 [containing 3.9 g of 2 (*N*-morpholino)ethanesulfonic acid, 0.048 g of magnesium sulfate, 0.066588 g of calcium chloride, 0.052824 g of diammonium hydrogen phosphate, 2 ml of tungstate/selenite solution (0.5 g of sodium hydroxide, 3 mg of sodium selenite, and 4 mg of sodium tungstate, dissolved in 1 liter of double distilled water, which was filtered with a 0.22-μm filter, stored at 4°C), and 2 ml of SL10 solution (10 ml of 25% hydrochloric acid, 1.5 g of ferrous chloride, 0.19 g of cobalt chloride, 0.1 g of manganese chloride, 0.07 g of zinc chloride, 0.062 g of boric acid, 0.036 g of sodium molybdic acid, 0.024 g of nickel chloride, and 0.017 g of copper chloride dissolved in 1 liter of double distilled water, which was filtered with a 0.22-μm filter, stored at 4°C), and 15.0 g of agar in 1 liter of pH 7.2 water] (73) for bacterial isolation. After the samples incubated at 25°C for 3 to 14 days, the different isolates according to morphological characteristics and colony size were selected. Single colonies from pure culture colonies were inoculated on LB media and stored at −20°C.

The 16S rRNA gene of each isolate was amplified with primers F27 (5′-AGTTTGATCMTGGCTCAG-3′)/R1492 (5′-GGTTACCTTGTTACGACTT-3′) (74). A total volume of 50 μl of PCR mixture contained 25 μl of 2× PCR *Taq* MasterMix, 2.0 μl of DNA templates, 2.0 μl of 10 μM forward primer F27, 2.0 μl of 10 μM reverse primer R1492, and 19 μl of H_2_O. PCR conditions were 2 min at 98°C; followed by 35 cycles of 10 seconds at 98°C, 15 seconds at 55°C, and 1 min at 72°C; and a final extension of 5 min at 72°C. PCR products were examined by electrophoresis on 1.5% agarose gels in 1× Tris-acetate-EDTA (TAE) buffer (40 mM Tris-acetate/1 mM EDTA [pH 8]) and sequenced by TsingKe (Kunming). Isolate taxonomy was determined through the Sequence Match function of the ribosomal database project (RDP) (71). For further exploration of the phylogenetic relationship between enriched genera from high-throughput sequencing and the isolates, similarity was shown by alignment of the sequence of enriched genera to isolates. We chose the significantly enriched isolates from the rhizosphere soil treated with ginsenosides to test their degradation activity.

### Autotoxic ginsenoside degradation ability of isolates.

Flask culturing was used to test the influence of specific bacterial isolates on the concentrations of autotoxic ginsenosides based on a previous study with some modifications (50). Briefly, isolates were inoculated in LB medium, and the cell density was adjusted by the addition of sterilized water, with the final optical density at 590 nm (OD_590_) reaching 0.4. Then, 1.0 ml of fresh bacterial suspensions was inoculated into 200 ml of liquid minimal media containing 1-g/liter crude ginsenosides and shaken at 150 rpm for 48 h, 96 h, and 144 h. The minimal medium supplemented with distilled water was set as the blank control. Then, the concentrations of R_1_, Rg_1_, Re, Rb_1_, and Rd in minimal media were determined by HPLC combined with standard ginsenosides as described above.

### Effects of ginsenosides on the growth and biofilm formation of isolate B36.

The effect of ginsenosides on the growth of isolate B36 was measured following a published procedure with some modifications (50). Briefly, B36 cells were cultured in LB medium, and the cell density was diluted with sterilized water to a final OD_590_ of 0.4. A drop of 20 μl of fresh bacterial suspensions and 180 μl of 1/10 LB media were added into 96-well microplates to reach the final concentrations of ginsenosides of 0, 0.001, 0.005, 0.01, 0.1, and 1.0 g/liter. After incubation at 28°C for 52 h, the absorbance at 590 nm was used to determine bacterial growth curves (OD_590_ reads) with a Versa Max microplate reader (Molecular Devices, Sunnyvale, CA, United States). Six replicates were used for each concentration.

Biofilm formation was measured by a method described previously (75). Briefly, aliquots (20 μl) of the diluted isolates were incubated anaerobically in the wells of 96-well microplates with ginsenosides (0, 0.001, 0.005, 0.01, 0.1, and 1.0 g/liter) for 52 h at 28°C. After the remaining bacterial culture in the wells was discarded, bacterial cells bound to the wells were washed gently twice with PBS, air dried, and then stained with 200 μl of 0.1% (wt/vol) crystal violet for 15 min. After being washed 4 times with PBS to remove excess dye, the cell-bound dye was eluted using 200 μl of 99% methanol. Biofilm formation was quantified by measuring the OD_490_.

### Effects of ginsenosides on the antagonistic activity of B36 against the soilborne pathogen.

The above-mentioned degradative isolate B36 was screened for its antagonistic activity against *I. destructans* isolated from infected sanqi roots following the method described in a previous study with some modifications (76). The pathogen mycelium block was obtained with a 6-mm-diameter hole punch and placed in the middle of the petri dish. Then, the degradative isolates were placed at the same distance around the pathogen mycelium block. The distance between the test isolates and the middle of the petri dish was 25 mm. Plates of only pathogen grown on potato dextrose agar medium (containing 200 g of potato, 20 g of glucose, and 15 g agar in 1 liter of water) were used as controls. Four replicate plates were used per treatment. All treatments were incubated at 25°C for 5 days. The radial growth of the pathogen was determined by measuring the colony semidiameter. The growth inhibition rate was calculated as follows:

growth inhibition rate (%)=100×(radial growth of control − radial growth of treated sample)/radial growth of control

### Evaluation of B36 to control root rot disease and colonize rhizosphere soil.

A pot experiment was performed to assess the ability of B36 to suppress soilborne disease and colonize the rhizosphere. Spontaneous rifampicin-resistant mutants of *Burkholderia* sp. B36 were obtained as described previously with some modifications (77). Briefly, colonies of the strain were transferred to tryptic soy agar (TSA) (containing 15.0 g of tryptone, 5.0 g of peptone, 5.0 g of sodium chloride, 5 mg of chloramphenicol, and 15.0 g of agar in 1 liter of pH 7.2 water) plates containing increasing concentrations (10, 50, 100, 150, and 200 μg/ml) of rifampicin and 5-μg/ml chloramphenicol. The resistant mutant was selected on TSA amended with rifampicin at a concentration of 200 μg/ml. The stability of rifampicin resistance in the mutants was confirmed, and the growth rate of the rifampicin-resistant mutants was found to be similar with that of their respective wild types. Eighty grams of soil from a pine forest was placed in the pot and sterilized at 121°C for 20 min. The soilborne pathogen *I. destructans* (10^6^ CFU/ml) and/or rifampicin-resistant mutant of B36 (OD_590_, 0.8) were added to the pot with sterilized pine soil. The pots with sterilized water were taken as the blank control. Ten surface-sterilized seeds were sown in each pot. Ten replicate pots were used per treatment. All pots were placed in a growth chamber with a photoperiod of 16 h light/8 h dark at 25°C. After 3 months of inoculation, the incidence rate of sanqi was measured. Incidence rate of sanqi (%) = 100 × (diseased seedlings/10). Then, the rhizosphere soils were collected for cell recovery counting according to a previous description with some modifications (72). Briefly, a total of 10 g of rhizosphere soil was added to 90 ml of sterilized water, which was shaken at 150 rpm for 30 min. The suspension was serially diluted 10-fold, 100-fold, and 1,000-fold with distilled water. Then, 100 μl of cell suspension from 3 different dilutions was plated onto TSA medium plates containing 200-μg/ml rifampicin and 5-μg/ml chloramphenicol. After an overnight culture at 35°C, the number of CFUs per gram of soil was determined. The experiment was conducted in triplicate.

### Statistical analysis.

SPSS version 17.0 software (SPSS Inc., Chicago, IL) and Prism 7.0 software (GraphPad Inc., USA) were used for general statistical analyses. Normality of distribution and homogeneity of variance were evaluated before a statistical analysis was conducted. Mean separation among treatments was analyzed by one-way analysis of variance (ANOVA) and Duncan’s multiple range test (*P < *0.05).

### Data availability.

All raw sequencing data have been submitted to the NCBI Sequence Read Archive (SRA) database under the accession number PRJNA751684.
