# Supplementary material for: Enrichment of Burkholderia in the Rhizosphere by Autotoxic Ginsenosides to Alleviate Negative Plant-Soil Feedback
Source: Microbiol Spectr. 2021 Nov 10;9(3):e01400-21. doi: 10.1128/Spectrum.01400-21 (PMC8579924; doi:10.1128/Spectrum.01400-21)
Supplement: SUPPLEMENTAL FILE 1 — Supplemental material. Download SPECTRUM01400-21_Supp_1_seq12.pdf, PDF file, 0.1 MB [file spectrum01400-21_supp_1_seq12.pdf]

Table S1 Processed sample data information to analyze bacterial community

Sample ID	Raw Tags	Clean Tags	Effective Tags	AvgLen(bp)
B1	199213	181398	171390	413
B2	216788	198332	192713	411
B3	198184	180876	173309	411
BG1	225992	203552	192496	413
BG2	235621	213988	202316	413
BG3	227119	205591	195998	412
RS1	194191	177575	175988	411
RS2	168595	153138	143638	413
RS3	192625	174981	164650	414
RSG1	185895	167576	155600	413
RSG2	156135	141170	130562	414
RSG3	219899	197340	185572	414
Sum	2420257	2195517	2084232	

Table S2 The relative abundance of Rhizobiales in different treatments.

Family	B	BG	RS	RSG
A0839	0.02±0.00	0.03±0.00	0.04±0.00	0.05±0.03
Beijerinckiaceae	0.83±0.03	1.05±0.08	1.03±0.07	1.17 ±0.02
Devosiaceae	0.57 ±0.09	0.64±0.02	0.93±0.19	1.13± 0.21
Hyphomicrobiaceae	0.08±0.02	0.10±0.01	0.16±0.02	0.21±0.01
Kaistiaceae	0.00±0.00	0.00±0.00	0.00±0.00	0.00±0.00
KF-JG30-B3	0.34±0.06	0.39±0.01	0.35±0.10	0.41±0.02
Labraceae	0.03±0.01	0.02±0.00	0.08± 0.03	0.06±0.01
Methyloligellaceae	0.06±0.01	0.09±0.01*	0.07±0.01	0.09±0.03
Methylopilaceae	0.00±0.00	0.00±0.00	0.00±0.00	0.00±0.00
Pleomorphomonadaceae	0.00±0.00	0.00±0.00	0.00±0.00	0.00±0.00
Rhizobiaceae	0.06±0.00	0.08±0.01	0.26±0.01	0.44±0.07*
Rhizobiales_Incertae_Sedis	0.10±0.01	0.12±0.01	0.18±0.03	0.17±0.04
Rhodomicrobiaceae	0.01±0.00	0.01±0.00*	0.01±0.00	0.01±0.00
Stappiaceae	0.00±0.00	0.00±0.00	0.00±0.00	0.00±0.00
uncultured_bacterium_o_Rhizobiales	0.22±0.02	0.21±0.00	0.25±0.01	0.31±0.04
Xanthobacteraceae	7.09± 0.16	8.86±0.31**	6.89±0.58	8.92±0.45

B represents the bulk soil. BG represents the bulk soil amended with exogenous ginsenosides. RS represents the rhizosphere soil of *P. notoginseng*. RSG represents the rhizosphere soil amended with exogenous ginsenosides. An asterisk (*) indicates that the differences between bulk or rhizosphere soil and its corresponding treatment with exogenous ginsenosides at $p < 0.05$.

Asterisks (**) indicates that the differences were significant at $p < 0.01$.

Table S3 The relative abundance of Betaproteobacteriales in different treatments.

Family	B	BG	RS	RSG
A21b	0.10±0.02	0.14±0.01	0.11±0.03	0.14±0.02
Aquaspirillaceae	0.00±0.00	0.00±0.00	0.00±0.00	0.00±0.00
B1-7BS	0.04±0.01	0.03±0.00	0.04±0.01	0.02±0.00
Burkholderiaceae	1.04±0.07	1.32±0.03*	2.59±0.13	4.95±0.46**
Gallionellaceae	0.00±0.00	0.00±0.00	0.00±0.00	0.00±0.00
Methylophilaceae	0.00±0.00	0.00±0.00	0.00±0.00	0.00±0.00
Neisseriaceae	0.00±0.00	0.00±0.00	0.00±0.00	0.00±0.00
Nitrosomonadaceae	0.64±0.02	0.86±0.01**	1.05±0.08	1.01±0.10
Rhodocyclaceae	0.00±0.00	0.00±0.00	0.00±0.00	0.02±0.01
SC-I-84	0.66±0.06	1.08±0.02**	0.78±0.14	0.91±0.04
TRA3-20	0.01±0.00	0.01±0.00	0.04±0.00	0.05±0.01

B represents the bulk soil. BG represents the bulk soil amended with exogenous ginsenosides. RS represents the rhizosphere soil of *P. notoginseng*. RSG represents the rhizosphere soil amended with exogenous ginsenosides. An asterisk (*) indicates that the differences between bulk or rhizosphere soil and its corresponding treatment with exogenous ginsenosides at $p < 0.05$. Asterisks (**) indicates that the differences were significant at $p < 0.01$.

Table S4 The relative abundance of Burkholderiaceae in different treatments.

Genus	B	BG	RS	RSG
Achromobacter	0.00±0.00	0.00±0.00	0.01±0.00	0.01±0.00
Bordetella	0.00±0.00	0.00±0.00*	0.02±0.01	0.03±0.01
Burkholderia-Caballeronia-Paraburkholderia	0.66±0.05	0.89±0.03*	1.43±0.19	3.24±0.44*
Caenimonas	0.01±0.00	0.00±0.00	0.01±0.01	0.01±0.00
Comamonas	0.00±0.00	0.00±0.00*	0.00±0.00	0.00±0.00
Cupriavidus	0.00±0.00	0.00±0.00	0.00±0.00	0.01±0.00
Duganella	0.00±0.00	0.00±0.00	0.00±0.00	0.00±0.00
Herbaspirillum	0.00±0.00	0.00±0.00	0.00±0.00	0.00±0.00
Herminiimonas	0.00±0.00	0.00±0.00	0.00±0.00	0.00±0.00
Hydrogenophaga	0.00±0.00	0.00±0.00	0.00±0.00	0.00±0.00
Ideonella	0.00±0.00	0.00±0.00*	0.00±0.00	0.00±0.00
Limnobacter	0.04±0.00	0.02±0.00**	0.01±0.00	0.03±0.01
Limnohabitans	0.00±0.00	0.00±0.00	0.00±0.00	0.00±0.00
Massilia	0.02±0.00	0.03±0.01	0.04±0.01	0.09±0.02
Methylibium	0.01±0.00	0.01±0.00	0.02±0.00	0.03±0.00
Mycoavidus	0.00±0.00	0.00±0.00	0.00±0.00	0.00±0.00
Noviherbaspirillum	0.01±0.00	0.00±0.00*	0.04±0.00	0.07±0.04
Pandoraea	0.00±0.00	0.00±0.00	0.00±0.00	0.01±0.00
Paralcaligenes	0.01±0.00	0.00±0.00*	0.00±0.00	0.02±0.00**
Paucibacter	0.00±0.00	0.00±0.00	0.00±0.00	0.00±0.00*
Pelomonas	0.00±0.00	0.00±0.00	0.00±0.00	0.00±0.00
Pigmentiphaga	0.00±0.00	0.00±0.00	0.00±0.00	0.00±0.00
Piscinibacter	0.00±0.00	0.00±0.00	0.00±0.00	0.01±0.00
Pseudorhodoferax	0.00±0.00	0.00±0.00	0.00±0.00	0.00±0.00
Ralstonia	0.00±0.00	0.00±0.00	0.00±0.00	0.02±0.00*
Ramlibacter	0.03±0.00	0.03±0.00	0.11±0.02	0.10±0.01
Rhizobacter	0.04±0.00	0.06±0.00*	0.07±0.01	0.09±0.01
uncultured_bacterium_f_Burkholderiaceae	0.19±0.01	0.23±0.01	0.63±0.01	1.06±0.08**
Undibacterium	0.00±0.00	0.00±0.00	0.01±0.00	0.01±0.00*
Variovorax	0.02±0.00	0.03±0.00	0.16±0.04	0.12±0.02

B represents the bulk soil. BG represents the bulk soil amended with exogenous ginsenosides. RS represents the rhizosphere soil of *P. notoginseng*. RSG represents the rhizosphere soil amended with exogenous ginsenosides. An asterisk (*) indicates that the differences between bulk or rhizosphere soil and its corresponding treatment with exogenous ginsenosides at $p < 0.05$. Asterisks (**) indicates that the differences were significant at $p < 0.01$.
